# Supplementary material for: Selective silencing of antibiotic-tethered ribosomes as a resistance mechanism against aminoglycosides
Source: Nat Commun. 2025 Oct 29;16:9568. doi: 10.1038/s41467-025-65298-7 (PMC12572303; doi:10.1038/s41467-025-65298-7)
Supplement: Supplementary file 1 — Supplementary Information [file 41467_2025_65298_MOESM1_ESM.pdf]

Supplementary Information for

Selective silencing of antibiotic-tethered ribosomes as a resistance mechanism against aminoglycosides

Nilanjan Ghosh Dastidar[1,2,†], Nicola S. Freyer[1,2,†], Valentyn Petrychenko[3], Ana C. de A. P. Schwarzer[3], Bee-Zen Peng[1], Ekaterina Samatova[1], Christina Kothe[1], Marlen Schmidt[4], Frank Peske[1], Antonio Z. Politi[5], Henning Urlaub[6,7], Niels Fischer[3], Marina V. Rodnina[1*], Ingo Wohlgemuth[2*]

[1]Department for Physical Biochemistry, Max Planck Institute for Multidisciplinary Sciences, Göttingen, Germany

[2]Project Group Fidelity of Protein Synthesis in vivo, Department for Physical Biochemistry, Max Planck Institute for Multidisciplinary Sciences, Göttingen, Germany

[3]Project Group Molecular Machines in Motion, Department for Physical Biochemistry, Max Planck Institute for Multidisciplinary Sciences, Göttingen, Germany

[4]Genetic Engineering Heidelberg GmbH, Heidelberg, Germany

[5]Facility for Light Microscopy, Max Planck Institute for Multidisciplinary Sciences, Göttingen, Germany

[6]Bioanalytical Mass Spectrometry Group, Max Planck Institute for Multidisciplinary Sciences, Göttingen, Germany

[7]Institute of Clinical Chemistry, Bioanalytics, University Medical Center Göttingen, Göttingen, Germany

[†]These authors contributed equally

[*]Correspondence: Ingo.Wohlgemuth@mpinat.mpg.de, Rodnina@mpinat.mpg.de

Supplementary Figures

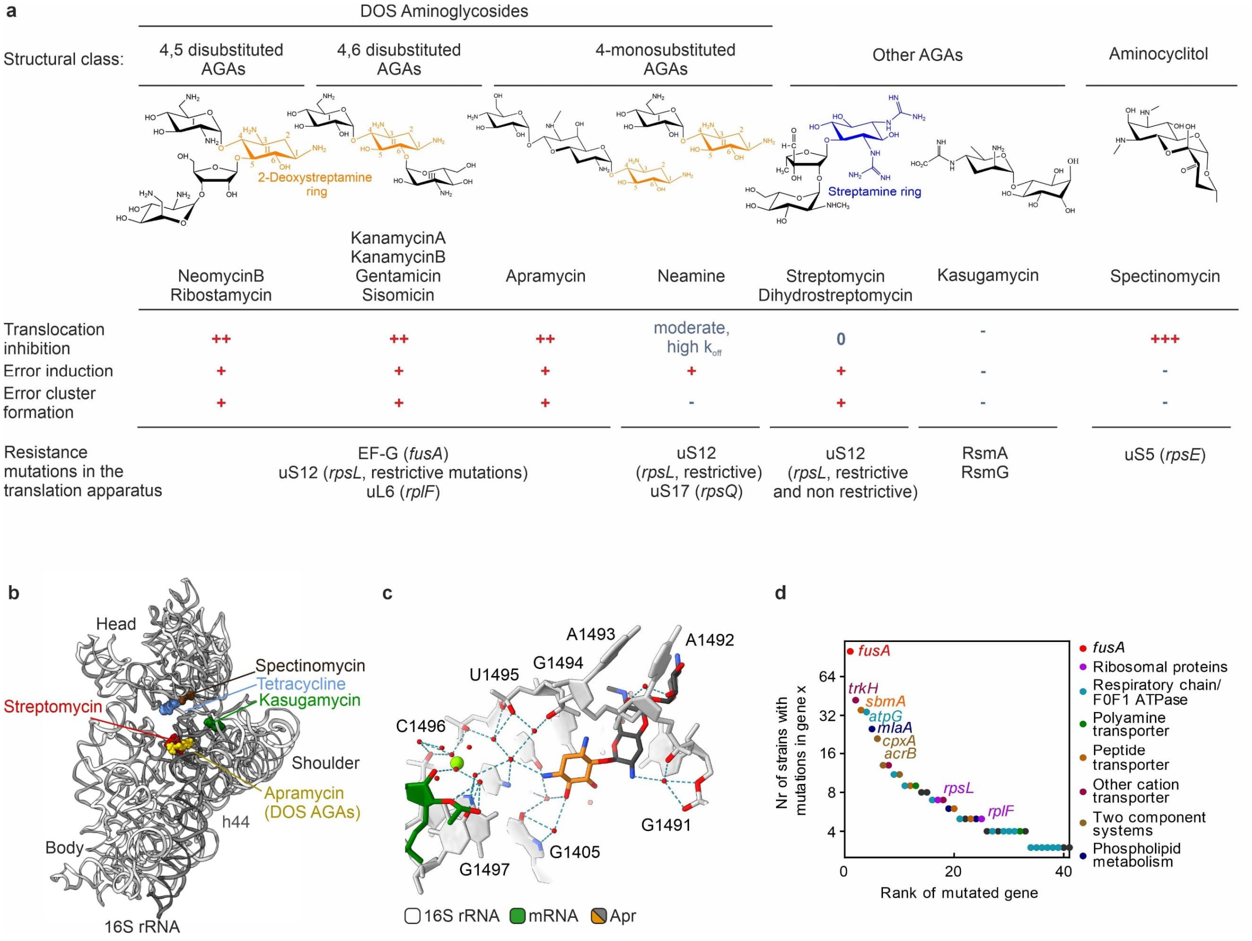

Supplementary Figure 1. Action of DOS aminoglycosides on the ribosome, related to Figure 1
(a) Chemical classification of aminoglycoside antibiotics (AGAs). Most AGAs belong to the class of 2-deoxystreptamine (2-DOS) AGAs. Neomycin (Neo) and ribostamycin (Rib) share a 4,5 disubstituted 2-DOS ring (II). Kanamycin (KanA, KanB), gentamicin (Gen) and sisomicin (Sis) belong to the 4,6 disubstituted 2-DOS family, whereas in apramycin (Apr) the 2-DOS ring is monosubstituted at position 4. All these AGAs target helix 44 of 16S rRNA at the decoding center (DC) of the ribosome. They induce misreading, resulting in single errors and error clusters [1], and slow down tRNA-mRNA translocation. Treatment with DOS AGAs promotes *fusA* (coding for EF-G) resistance mutations in many pathogens [2,3,4,5,6,7,8,9,10,11,12,13,14,15,16,17,18,19,20,21,22,23,24,25,26,27,28,29,30,31,32,33,34,35,36,37,38,39,40,41]. Less frequently, mutations are found in *rpsL* (coding for uS12) and *rplF* (coding for uL6) [42]. Neamine (Nea) is the smallest 2-DOS AGA with only two rings that are necessary to recognize the binding site in the DC. It has a lower affinity to the ribosome [43], higher Minimal Inhibitory Concentrations (MIC) [44], and does not promote error cluster formation [1]. Neamine resistance mutations were reported in *rpsL* and *rpsQ* (coding for uS17) [45,46,47]. Streptomycin (Str) and dihydrostreptomycin (Dhs) have a streptamine ring; they induce misreading and error cluster formation [1] but have only a moderate effect on translocation [48] and induce resistance mutations in *rpsL* [12]. Non-restrictive resistance mutations in uS12 prevent drug binding, confer high Str resistance and are associated with moderate fitness costs. In contrast, restrictive *rpsL* mutations confer a higher translation fidelity than the wt but only moderate resistance, and have higher fitness costs.

Kasugamycin (Ksg) is a small, structurally unrelated AGA that inhibits initiation [49] and the transition to elongation in a sequence-dependent manner [50]. Resistance mutations against Ksg are selected in the methyltransferases RsmA and RsmG. Spectinomycin (Spc) is structurally similar to AGAs and belongs to the class of aminocyclitols; it inhibits translocation but has no effect on the decoding fidelity of the ribosome. Spc resistance mutations evolve in *rpsE* (encoding uS5) [12,51,52]. *FusA* mutations confer little or no resistance against Nea, Str or Spc (Figure 1C) [24,53], consistent with the fact that *fusA* mutations are only selected upon 2-DOS-AGA treatment [12].
(b) Superposition of drug binding sites on the small ribosomal subunit (SSU). Shown is the binding site for Apr (gold) and other 2-DOS AGAs at the top of helix 44 of 16S rRNA (PDB entry 7PJS) [54]. At elevated drug concentrations 2-DOS AGAs can also bind to secondary binding sites, which synergistically inhibits translation [54]. However, because rRNA mutations in helix 44 alone confer high AGA resistance by preventing AGA binding [44], secondary binding sites are less important for AGA uptake and misreading and are not considered here. Unlike the 2-DOS AGAs, Str interacts with the phosphate backbones of helices 44, 27 and 18 of 16S rRNA as well as the ribosomal protein S12; the latter is the hotspot for Str resistance mutations (PDB entry 8cgj) [55]. Ksg binds within the mRNA channel (PDB entry 8CEP) [55]. Spc binds to the end of helix 34 of 16S rRNA at the neck region of the SSU (PDB entry 8ca7) [55]. The nonaminoglycoside antibiotic tetracycline binds to the minor groove of helix 34 and the loop of helix 34 (PDB entry 8cf1) [55].
(c) Apr (dark grey with DOS ring orange) primary binding site at the top of helix 44 (PDB entry 7PJS) [54]. DOS rings I and II are essential for the decoding site recognition and the stabilization of the error-prone conformation of the ribosome.
(d) Rank plot of genes with resistance mutations evolved in laboratory evolution experiments upon treatment with 2-DOS AGAs. Mutational analysis of 108 AGA-treated *Escherichia coli* strains were retrieved from the literature [2,3,4,5,6,7,8,12,14,15,16]. Categories of mutated genes are indicated. *fusA* is by far the most sampled gene in laboratory evolution experiments.

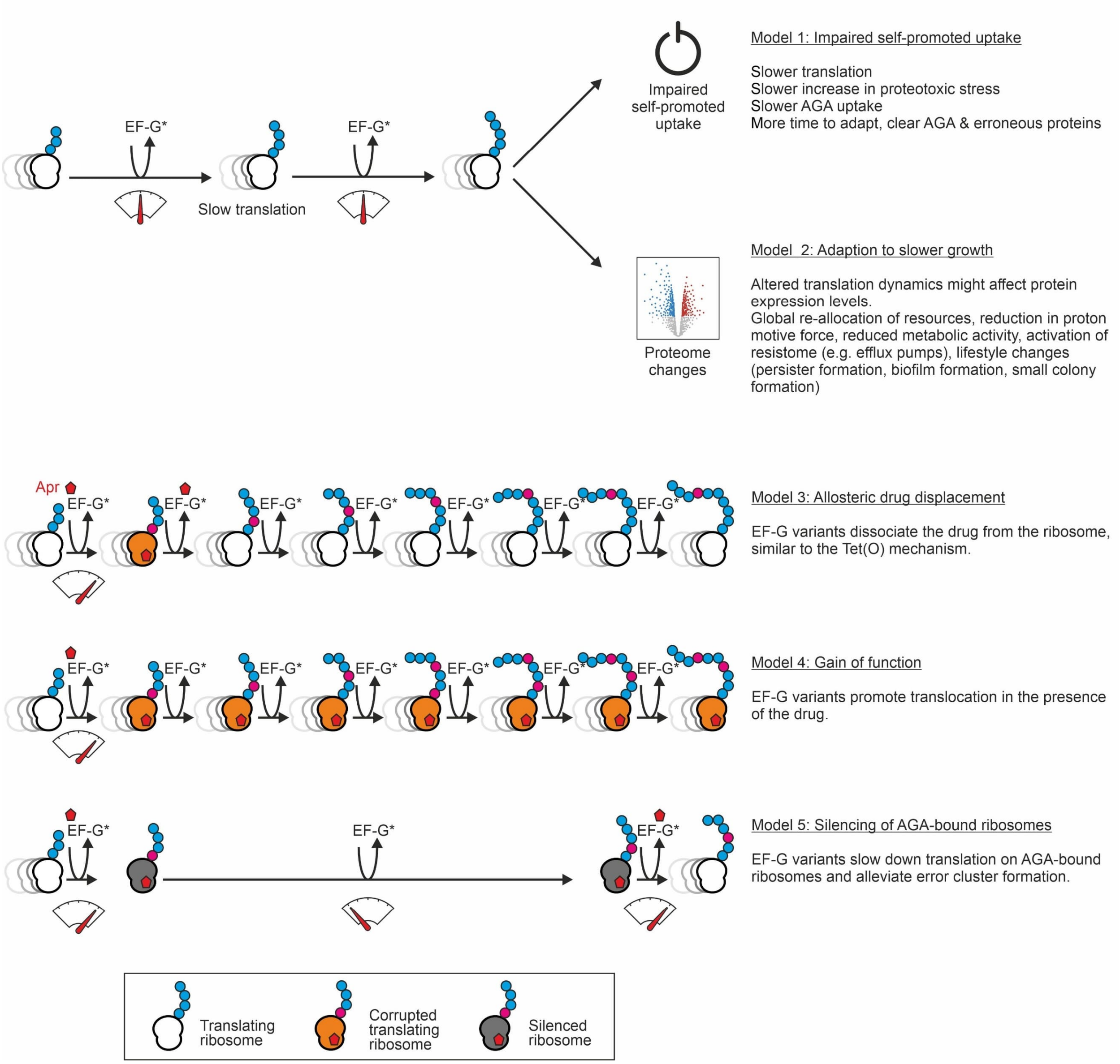

Supplementary Figure 2. Potential models of *fusA*-mediated resistance mechanisms, related to Figure 1 Models 1 and 2 suggest that EF-G resistance variants could indirectly contribute to resistance by affecting translation velocity. While the effect of EF-G resistant variants on translation have not been tested so far, at least two different models of resistance can be envisioned:

<u>Model 1. Direct slowdown of self-promoted AGA uptake.</u> If AGA-induced misreading is unchanged, slower translation with mutant EF-G might lead to a slower accumulation of faulty proteins. As a result, quality control machinery and efflux pumps could better cope with proteotoxic stress, resulting in a slower AGA uptake [56,57,58] and allowing cells to grow at otherwise lethal concentrations.

<u>Model 2. Adaption to slower translation.</u> Slower translation might change the proteome composition. Such changes could directly contribute to the resistome, for example, in *Pseudomonas aeruginosa*, *fusA1* mutations were reported to lead to elevated levels of mexXY efflux pumps and Type III secretion system (T3SS) [28]. However, the AGA resistance correlates poorly with mexXY expression levels [59]. In *Salmonella enterica*, *fusA* mutations were associated with lower levels of HemA, the enzyme which catalyzes the first committed step in heme biosynthesis, resulting in lower levels of respiration [60], which is often associated

with higher AGA resistance. Alternatively, *fusA* mutations could indirectly render bacteria resistant by changes in the re-allocation of resources and metabolic fluxes, e.g., *fusA* mutations were proposed to disturb the levels of ppGpp [61], a global transcription and translation regulator often associated with antibiotic resistance [62].

Alternatively, models 3-5 suggest that EF-G resistance variants could directly interfere with AGA action on the ribosome:

Model 3. Allosteric displacement of the drug. None of the amino acid substitutions in EF-G are in close proximity to AGA on the ribosome, making the direct displacement of the drug unlikely. However, EF-G variants have been proposed to induce allosteric changes that lower the affinity of the drug to the ribosome [8,24], similar to the mechanism of the tetracycline resistance protein Tet(O), an EF-G homolog, which uses its GTPase activity to remove tetracycline from the A site of the ribosome [63].

Model 4. Gain of function. EF-G variants were suggested to facilitate translocation in the presence of AGA [11], although such gain-of-function mechanism seems unlikely considering the widespread distribution of resistance mutations across EF-G.

Model 5. Ribosome silencing mechanism. EF-G variants could affect the error load in the AGA-affected cell. While EF-G variants do not restore the fidelity of AGA-bound ribosomes, because EF-G is not bound to the ribosome during decoding, they may affect formation of error clusters. If EF-G mutants slow down translation, AGA-bound ribosomes would perform fewer elongation cycles before the drug dissociates. Both the allosteric displacement (model 3) and the translation deceleration (model 5) could in principle alleviate error cluster formation and thus lower the proteotoxic burden that drives the self-promoted AGA uptake.

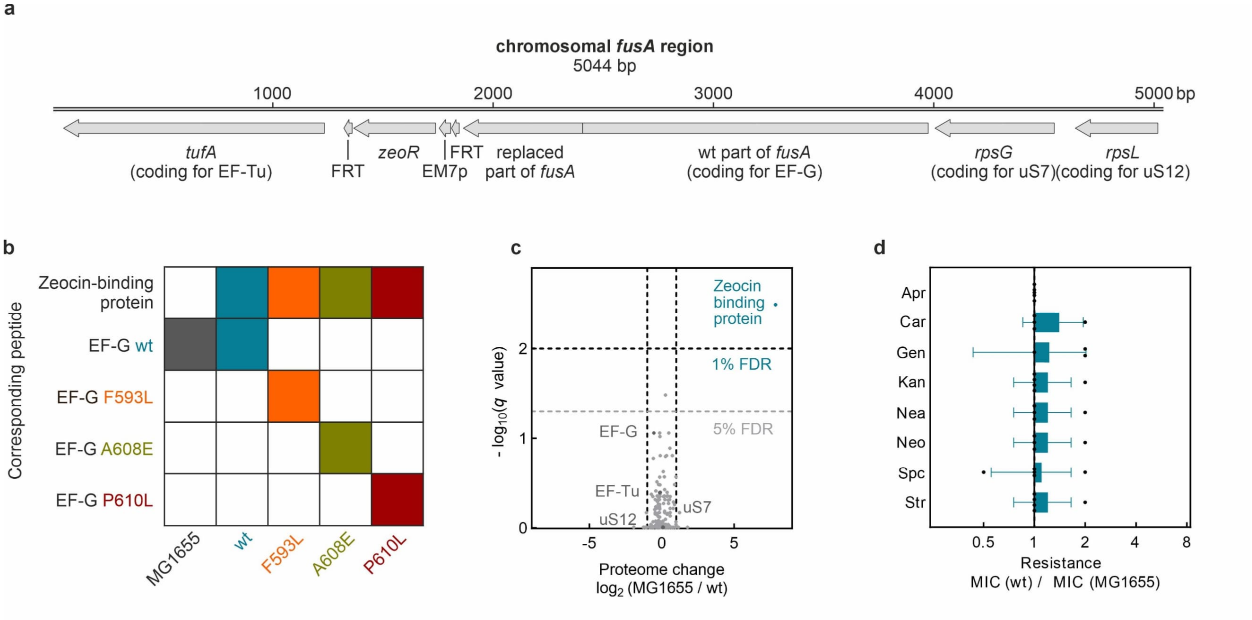

Supplementary Figure 3. Generation and characterization of chromosomal *fusA* mutants, related to Figure 1

(a) Schematic of the operon structure after insertion of *fusA* mutations. Correct insertion was confirmed by DNA sequencing.

(b) Targeted MS-based detection of EF-G variants. Each row corresponds to a particular peptide (Zeocin-binding protein, wt EF-G, or EF-G variant carrying one of the indicated mutations), while each column represents an *E. coli* strain (MG1655, wt, F593L, A608E, and P610L). A colored square indicates that the corresponding peptide was detected in that strain. Revertants or cross contaminations were not observed. The analysis confirms identity of the strains on the protein level.

(c) Proteome comparison of wt and MG1655 by quantitative MS (data independent acquisition). The volcano plot represents $\log_2$-fold changes in protein abundance (X-axis) and negative $\log_{10}$ adjusted (Benjamini-Hochberg) *P* values (Y-axis) for the parental strain MG1655 to the wt strain (n=4 biological replicates). Apart from the expression of the introduced zeocin resistance protein, the cloning strategy did not introduce significant proteomic changes. Notably, expression levels of EF-Tu, uS7 and uS12, which are members of the same operon, are not significantly altered.

(d) Comparison of the drug susceptibility of wt and MG1655. The minimal inhibitory concentrations (MIC) of all drugs tested are not significantly altered. Bars represent the mean ± SD of 5 biological replicates (n=5).

a

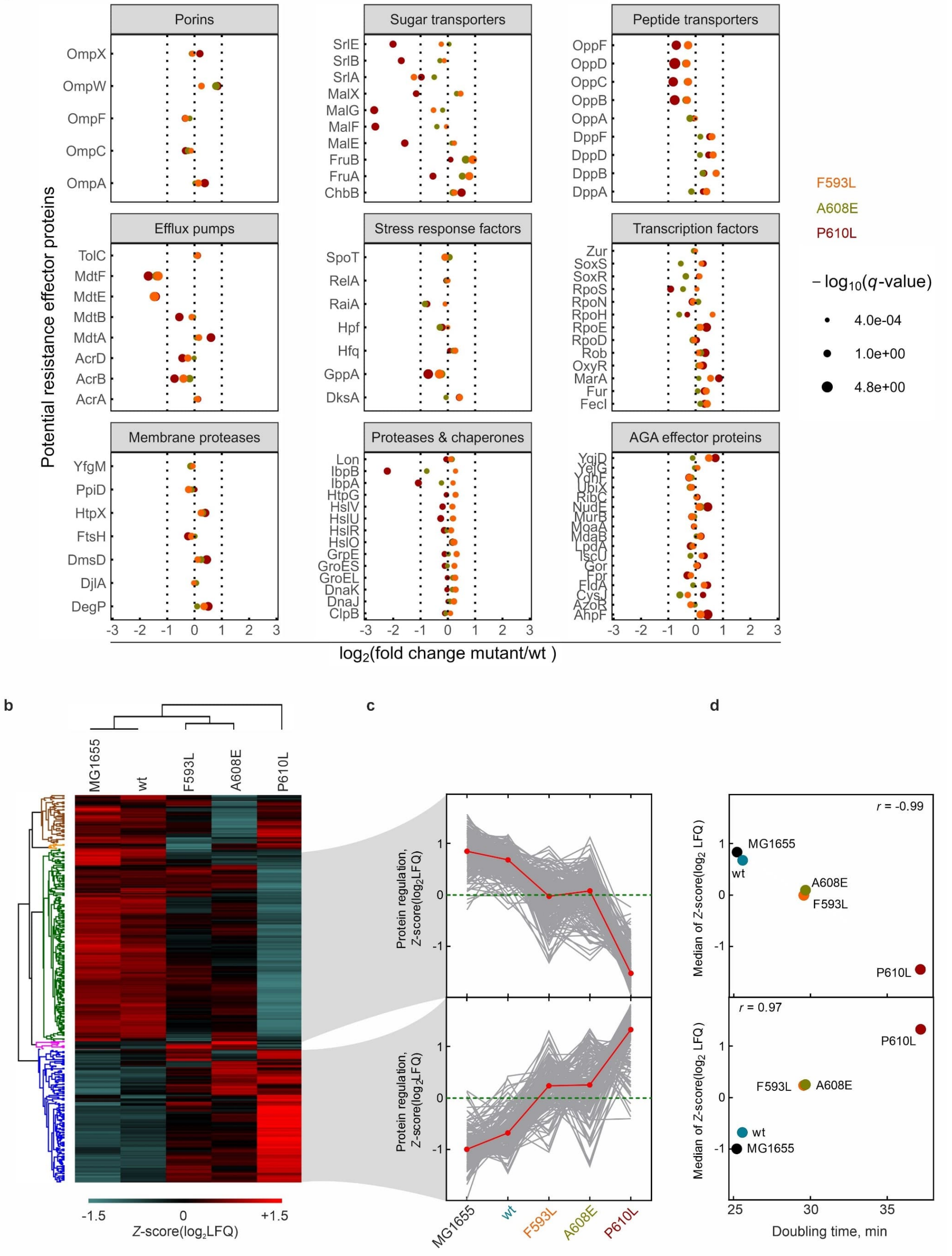

e

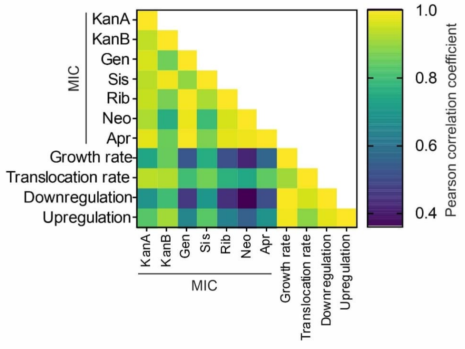

f

Metabolic pathway	Enrichment factor	Benj. Hoch. FDR
L-tryptophan biosynthesis	15.5	1.9×10^{-3}
L-leucine biosynthesis	15.5	1.9×10^{-3}
L-isoleucine biosynthesis	9.3	9.9×10^{-3}

Supplementary Figure 4. Proteome changes induced by *fusA* mutations, related to Figure 1

(a) Subset of data from Figure 1D showing the regulation of proteins involved in AGA action or resistance. No systematic upregulation of porins or transporters that facilitate AGA entry into bacterial cell was observed [64]. Efflux pumps, including AcrA, AcrD, TolC (RND transporters), which promote AGA efflux in *E. coli* [65,66] were also not upregulated. Proteins involved in ppGpp-responsive regulation —which could potentially confer resistance via global reallocation of resources and major transcription factors orchestrating global stress responses—showed no significant changes. Similarly, chaperones and proteases linked to proteostasis [67,68,69] were not systematically upregulated. Finally, proteins previously identified in systematic screens as conferring AGA resistance upon overexpression [70] showed no consistent upregulation. These findings indicate that none of the known AGA resistance pathways are activated in *fusA* mutants.

(b) Hierarchical clustering of significantly regulated proteins in MG1655, wt, and *fusA* mutants. Significantly regulated proteins were identified by multiple sample ANOVA test and compared by their *Z*-scores. Biological replicates (n = 4) were averaged. Proteins and strains were hierarchically clustered by their Euclidean distance and proteins were grouped in 5 clusters of co-regulated proteins. In the three minor clusters (saddle brown, dark orange, and magenta), proteins shared no common regulation pattern between *fusA* mutants and thus these proteins cannot constitute a common resistance mechanism. In contrast, in the two major clusters proteins were consistently down- (dark green, 234 proteins) or upregulated (blue, 163 proteins) in *fusA* mutant strains.

(c) Profile view of changes in expression levels of downregulated (upper panel) and upregulated (lower panel) proteins.

(d) Pearson correlation of cell growth and proteome adaptations. While overall the *fusA* mutants show only minor changes in their proteomes relative to the wt (Figure 1D), subtle adaptations of protein levels in the major clusters detected by hierarchical clustering correlate well with the growth rate differences (upper panel, $P = 0.0002$; lower panel: $P = 0.0039$).

(e) Correlation matrix of Pearson coefficients. Mutual correlation analysis of cell growth (Figure 1b), MIC values (Figure 1c), translocation rates (Figure 2a), and proteome regulation patterns (Figure S4b-d) across wt and *fusA* mutants (e.g. see Figures S4d, S5a). MIC values of different AGAs showed strong correlation, consistent with the idea that different *fusA* mutations confer resistance through a common mechanism. Cell growth, translocation rates, and proteome regulation patterns also correlate well, supporting the notion that proteome changes reflect subtle adaptations to the slower translocation rates and the resulting reduced growth. However, poor correlation between MICs values and growth-related metrics indicates that translocation rate differences, growth and proteome differences alone are not the primary drivers of AGA resistance.

(f) Pathway enrichment analysis of protein clusters. Pathway enrichment analysis of the five protein clusters was performed using Fisher's exact test, with false discovery rate (FDR) correction by the Benjamini-Hochberg procedure. Members of only three pathways were significantly enriched in the clusters, all related to amino acid biosynthesis and upregulated in response to slower translation, which triggers translation attenuation mechanisms. These findings suggest that changes in translation rate drive misadaptive responses typically observed under starvation conditions, but do not explain the observed resistance pattern (Figure S4e).

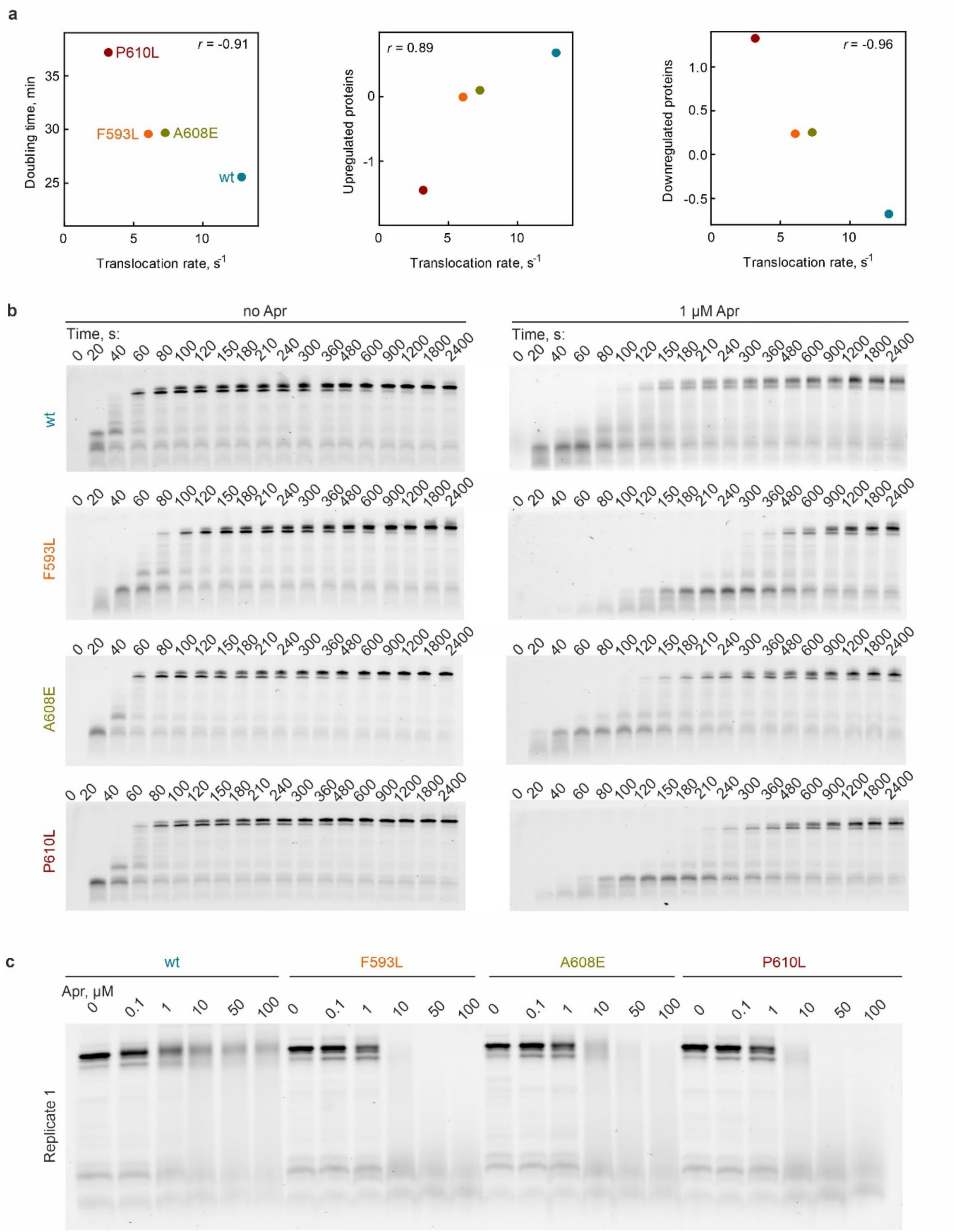

Supplementary Figure 5. Effect of EF-G variants and Apr on single-codon translocation and translation in vitro, related to Figure 2

(a) Correlation of the translocation rates measured in single-codon assay with growth rates (left panel) and proteome changes (center and right panel) for wt EF-G (blue symbol), F593L (orange), A608E (olive), and P610L (red). Data replotted from Figures 1b, 2a and S4b-d. Pearson coefficients as indicated.

(b) Time courses of *slyD* in-vitro translation with EF-G variants in the absence and presence of Apr (1 µM).
(c) Dependence of SlyD formation on Apr concentration. Full length product was quantified after 30 min of translation in the absence and presence of increasing Apr concentrations.

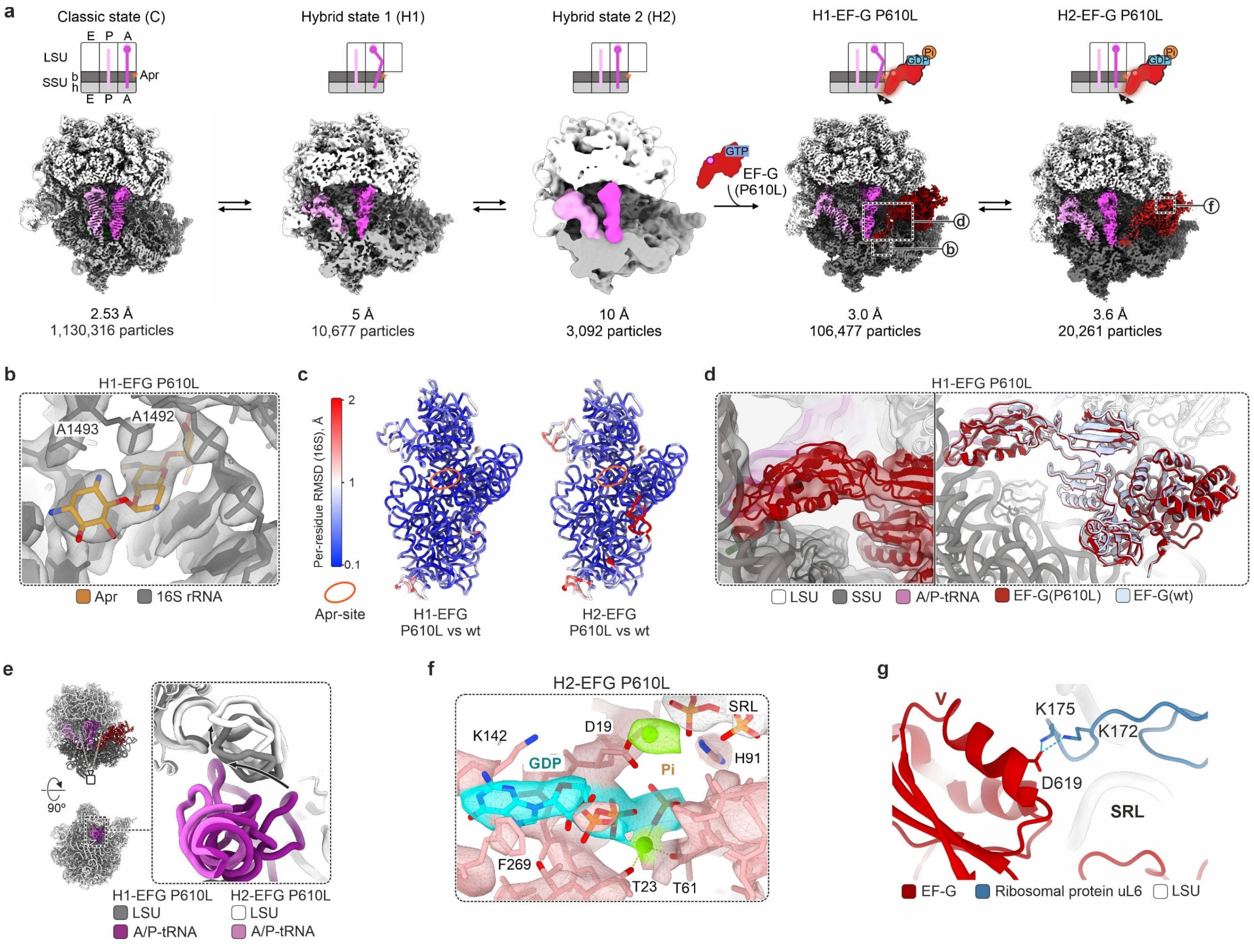

Supplementary Figure 6. Structural dynamics of EF-G P610L-driven translocation in presence of Apr, related to Figure 3

(a) Cryo-EM analysis of EF-G P610L-promoted translocation in the presence of Apr. Top: Schematics of tRNA positions. The arrows indicate dynamics of the tip of EF-G domain IV, similar to that observed for EF-G wt [54]. Bottom: Cryo-EM maps with respective overall resolution in Å and number of cryo-EM particle images. In Figure 3a-d, hybrid state (H) refers to the major tRNA hybrid state H1, while in Figure 3e hybrid state (H) summarizes H1 and H2 particle populations.
(b) Apr bound in the decoding center of the ribosomal complex with EF-G P610L and GDP-Pi in state H1.
(c) Per-residue RMSDs of 16S rRNA between EF-G P610L- and wt-bound complexes, revealing generally negligible structural differences, particularly at the Apr-binding site (orange circle). H1-EF-G wt: PDB 7PJV; H2-EF-G wt: PDB 7PJW [54].
(d) Structure of EF-G P610L-GDP-Pi in H1. Left: Cryo-EM density for domain IV, low-pass filtered to local resolution (6 Å). Right: Superposition with wt EF-G-GDP-Pi in H1 state (PDB 7PJW [54]), based on alignment by 23S rRNA.
(e) Repositioning of the elbow of A/P tRNA and helix 38 of 23S rRNA (H38) upon transition between tRNA hybrid states.
(f) Nucleotide binding pocket of EF-G P610L-GDP-Pi in H2.

(g) Interaction of LSU protein uL6 with domain V of EF-G. In the fusidic acid stalled ribosome-EF-G complex, residues K172 and K175 of uL6 form salt-bridges with D619 of EF-G (PDB 4V5F [71]); residues numbered according to *Thermus thermophilus* components here.

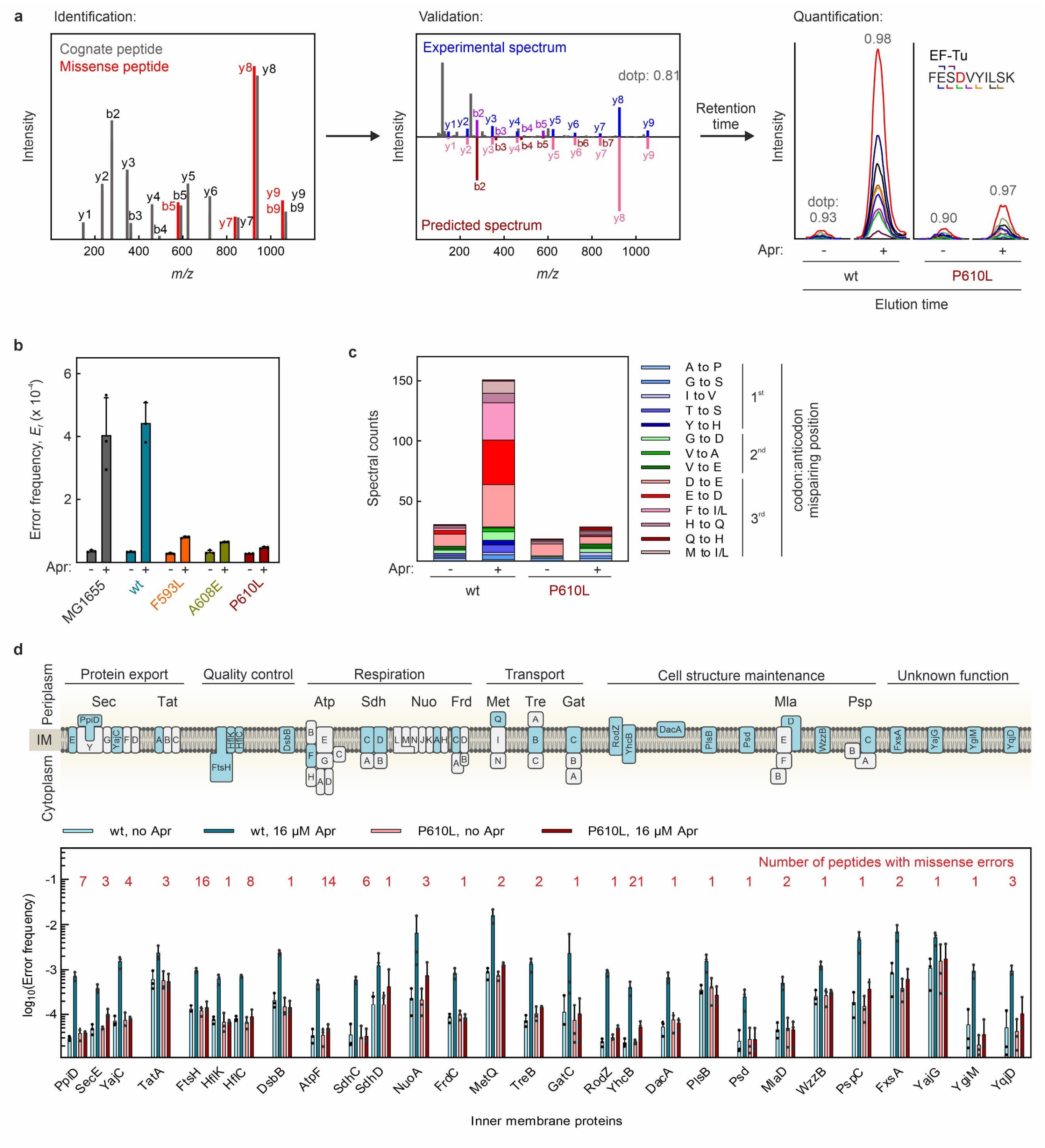

Supplementary Figure 7. Preservation of proteome integrity in *fusA* mutant strains, related to Figure 4
(a) Workflow for the identification, validation, and quantification of missense peptides with individual amino acid substitutions. Using the MaxQuant Dependent Peptide feature [72], missense peptide candidates are identified by comparing MS/MS spectra of correct and missense peptides ensuring the confident localization of the amino acid substitution (shown is a peptide with the substitution E308D, corresponding to a -14 delta mass). Missense peptide candidates are validated by matching their MS/MS spectra with fragmentation spectra predicted *in-silico* by the deep neural network Prosit [73]. Established fragmentation pattern and chromatographic retention times are used to quantify missense peptides across various biological states by label-free Parallel Reaction Monitoring (PRM). PRM traces are extracted

in Skyline [74]. Extracted fragment ions are color coded; amino acid substitutions indicated in red. Peptide abundances are calculated by integrating the signal of their co-eluting peptide fragments and error frequencies are estimated as the ratio of missense peptide and correct peptides abundances (see Methods).
(b) *fusA* mutations prevent Apr-induced (16 µM) translational misreading. Bars represent the mean error frequencies ± SD of 3 biological replicates (n=3) of the median of 28 misreading events.
(c) Semiquantitative analysis of the error burden in the proteome through spectral counting. Untreated and Apr-treated wt and P610L cells were lysed and proteins fractionated by SDS-PAGE. Missense peptide identifications in various proteins were counted (detection and identification as described above). Only types of substitutions whose counts are induced > 3-fold by AGA treatment were included. Position of the mismatch in the codon-anticodon interaction as indicated.
(d) Error frequencies in inner membrane proteins in the absence and presence of Apr (16 µM, 60 min). Error frequencies of 109 misreading events in 28 proteins were determined by label free PRM. Bars represent mean error frequencies ± SD of three biological replicates (n=3) of the medians of 1-21 misreading events per protein, as indicated above the bars.

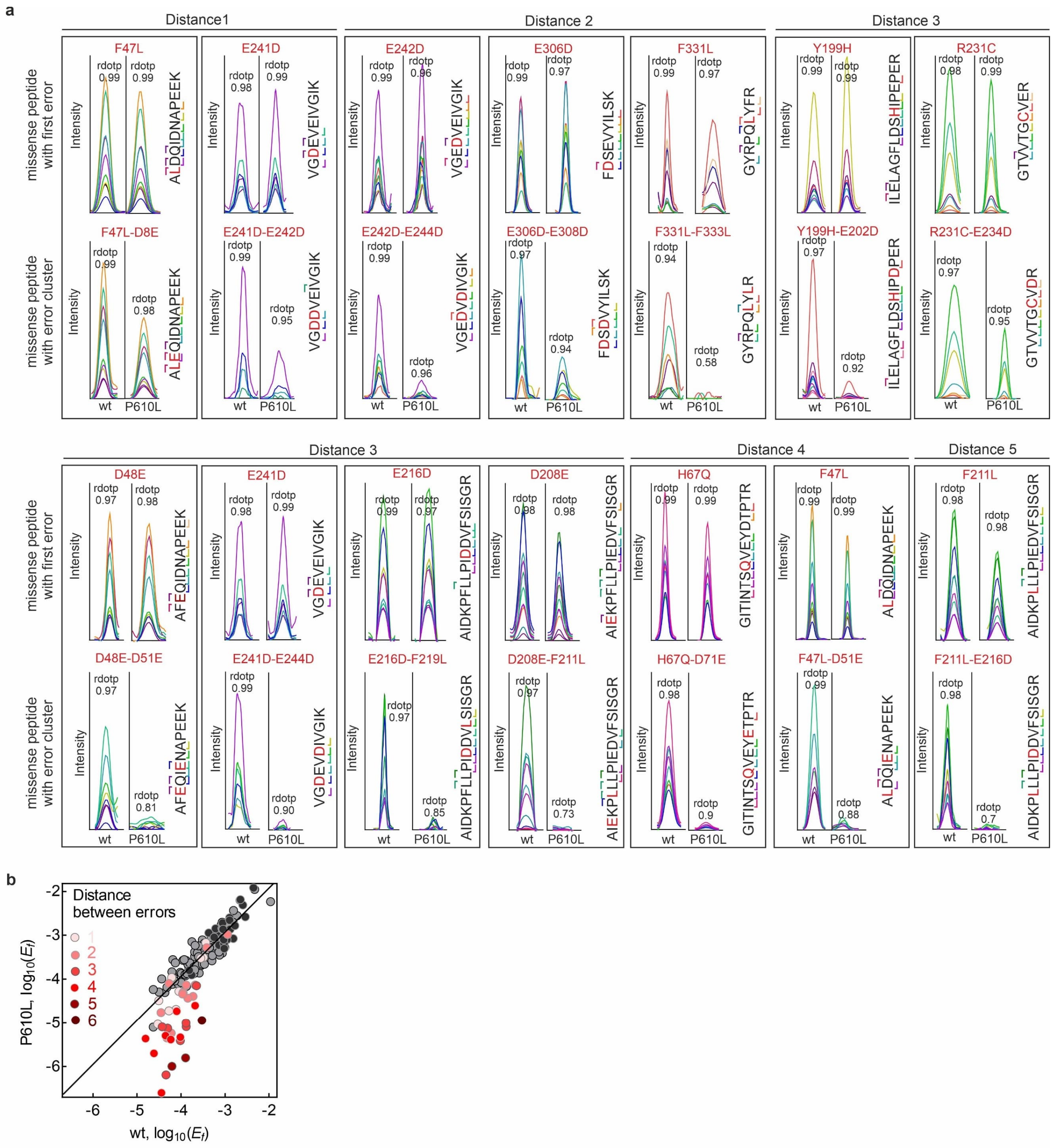

Supplementary Figure 8. *fusA* mutations prevent error cluster formation, related to Figure 5

(a) Chromatograms of eluting missense peptides with first errors and corresponding error clusters at induced conditions (wt: at 8-16 µM Apr; P610L: at 128-256 µM Apr; data are shown in Figure 5a,c). The conditions were chosen to ensure similar levels of single substituted peptides in wt and P610L. Extracted fragment ions are color-coded; amino acid substitutions indicated in red. Missense peptides were identified by the high sequence coverage, the coelution and identical fragmentation as of the spiked-in isotope-labeled reference peptides (ratio dot products ≥ 0.97). While missense peptides with short error clusters are often high abundant in wt and P610L cells, error clusters with more amino acid residues between the consecutive errors were strongly suppressed in P610L. To study this length effect in detail

we extended set of error clusters from 14 to 38 in Figures 5d and S8b. Plots represent the MS2 intensities of peptide fragments (Y-axis) over the retention time (X-axis).

(b) Comparison of the error load with single errors and error clusters between wt and P610L at conditions of maximal misreading (wt at 16 µM Apr, P610L at 256 µM Apr). Missense peptides with error clusters are less abundant in P610L than in wt cells (red circles, quantified by PRM). This effect depends on the distance between the first and the consecutive amino acid substitution (see replot in Figure 5d). In contrast to error cluster formation, EF-G (P610L) had no significant effect on other misreading events: Grey circles, single errors quantified by MS1-filtering in DDA runs; black circles, single errors quantified by targeted MS (PRM). The type of data acquisition had no impact on this conclusion. This shows that *fusA* mutations prevent error cluster formation, but have otherwise no significant impact on the proteome integrity indicating that the intrinsic ribosome fidelity is unaltered, consistent with the fact that EF-G does not affect decoding per se. Because error frequencies are influenced by the concentrations of competing aminoacyl-tRNAs [75], this also suggests that stoichiometries of cellular tRNAs are not substantially altered.

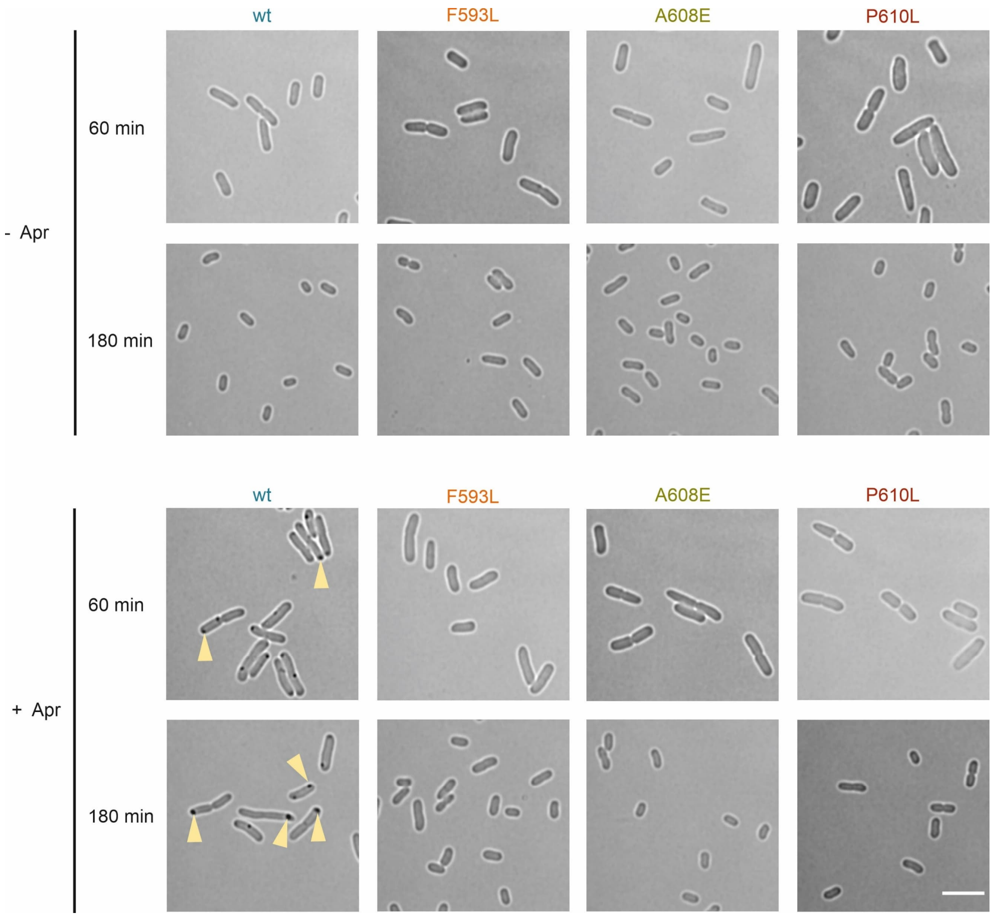

Supplementary Figure 9. *FusA* mutations prevent protein aggregation in vivo, related to Figure 6
Upper panel: brightfield images of wt and *fusA* mutant cells after 60 min and 180 min in the absence of Apr. Lower panel: same for cells after 60 min and 180 min of Apr treatment (16 µM). Images for 180 min time point same as Figure 6a. Yellow arrows point to inclusion bodies. Scale bar, 5 µm.

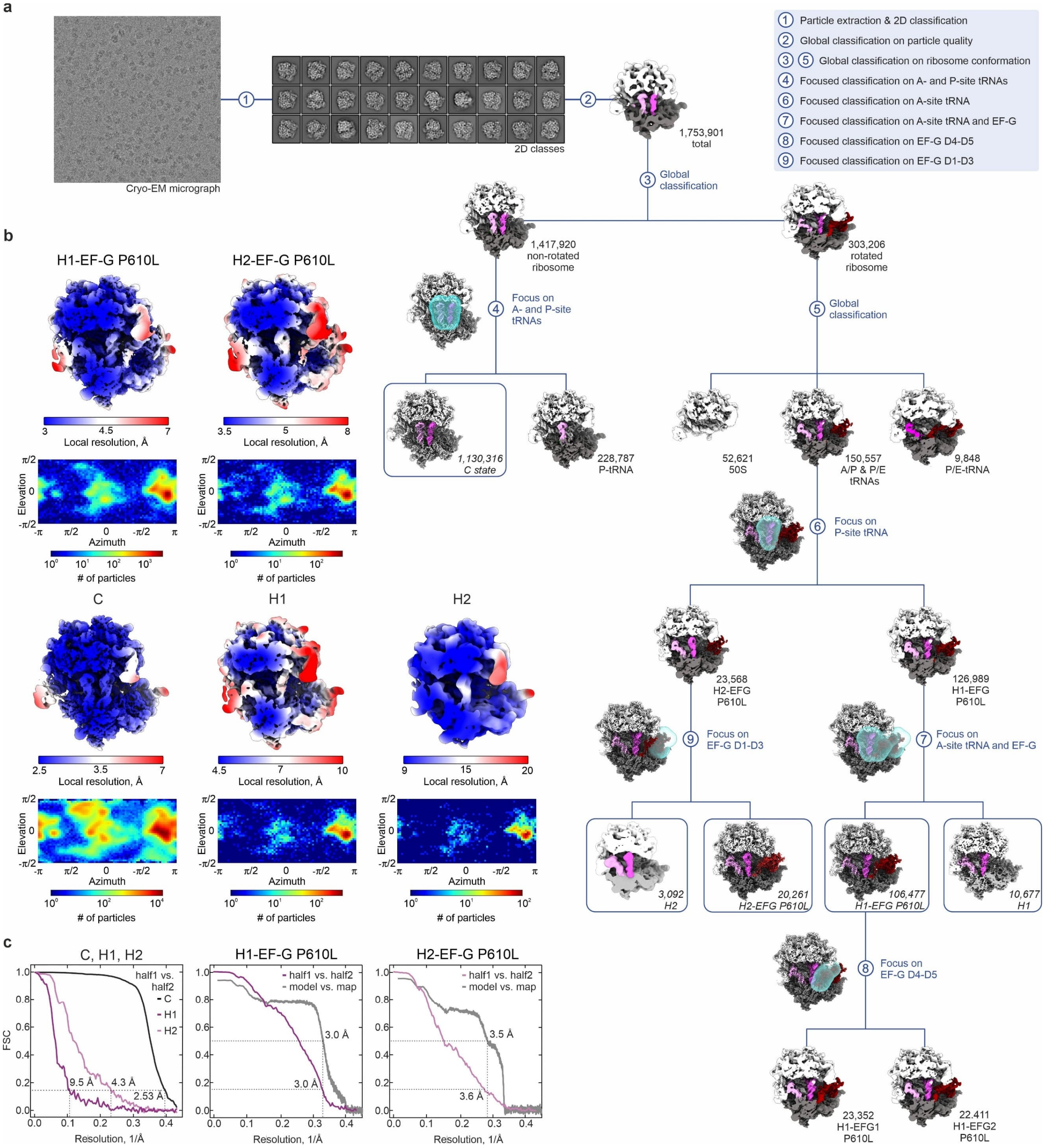

Supplementary Figure 10. Cryo-EM analysis, related to Methods

(a) Multi-step sorting of cryo-EM data with exemplary micrograph 2D classes and 3D classes together with masks used for focused classification. See Methods for details.

(b) Local resolutions and angular distributions of final cryo-EM maps.

(c) Fourier-Shell-Correlation curves of final cryo-EM reconstructions, computed between half-maps (half1 vs. half2), and between full maps and atomic models (model vs. map), respectively. Atomic models were built for EF-G P610L-bound complexes only.

Supplementary Figures

Supplementary Table 1. Cryo-EM structure determination

**Ribosomal complex**	H1–EF-G P610L	H2–EF-G(P610L)
Ribosomal state	tRNA hybrid state 1 with EF-G P610L in GDP-Pi form	tRNA hybrid state 2 with EF-G P610L in GDP-Pi form
**Database entries**		
EMDB ID	**EMD-54253**	**EMD-54254**
PDB ID	**9RTU**	**9RTV**
**Data collection**		
Microscope	Titan Krios	Titan Krios
Camera	Falcon III	Falcon III
Magnification	59,000	59,000
Voltage (kV)	300	300
Electron dose ($e^{-}/Å^2$)	30	30
Defocus range (μm)	0.2-1.5	0.2-1.5
Pixel size (Å)	1.16	1.16
**Cryo-EM reconstruction**		
Final particles (no.)	106,477	20,261
Point group symmetry	*C1*	*C1*
FSC-threshold	0.143	0.143
Resolution◊ (Å)	3.0	3.6
Resolution metric	gold standard FSC	gold standard FSC
**Atomic model refinement***		
Resolution§ (Å)	3.0	3.5
Cumulative RSCC (%) >0.8/>0.6/>0.4	0.44/0.89/0.95	0.40/0.89/0.96
Initial models used	PDB 7PJV	PDB 7PJW
Molprobity score	1.64	1.74
Clashscore	4.83	6.08
**No. Atoms/No. Residues/RSCC**		
Total	0.75	0.74
Protein	0.72	0.71
Nucleic acids	0.78	0.77
EF-G	0.63	0.60
**B-factors**		
Protein	37.60	47.56
Nucleotide	31.92	54.35
Ligands, Ions	18.84	34.55
**R.m.s. deviations**		
Bond lengths (Å)	0.005	0.005
Bond angles (°)	0.644	0.978
**Ramachandran plot**		
Favored (%)	94.21	93.89
Allowed (%)	5.79	6.09
Disallowed (%)	0.00	0.02

◊Resolution used for atomic model refinement and interpretation.
*For model refinement, the high-resolution H1–EF-G P610L map was resampled to 512×512×512 pixels, corresponding to a pixel size of 0.6525Å
§Resolution at which map-model FSC=0.5.

Supplementary Table 2. Resources

REAGENT or RESOURCE	SOURCE	IDENTIFIER
**Strains**		
MG1655	Leibniz Institute DSMZ	DSM 18039
BL21(DE3)	Thermo Fisher Scientific	EC0114
Engineered *fusA* strains (parental strain MG1655)	Genetic engineering Heidelberg	This manuscript
**Plasmids & Recombinant DNA**		
pET-24a vector, Kan resistance	Novagen	
pRed/ET	Genetic Engineering Heidelberg	
pET-24a (*fusA*_wt), *fusA* sequence derived from MG1655		This manuscript
pET-24a (*fusA*_G117C)	based on pET-24a (*fusA*_wt)	This manuscript
pET-24a (*fusA*_R371L)	based on pET-24a (*fusA*_wt)	This manuscript
pET-24a (*fusA*_F593C)	based on pET-24a (*fusA*_wt)	This manuscript
pET-24a (*fusA*_F593L)	based on pET-24a (*fusA*_wt)	This manuscript
pET-24a (*fusA*_F605I)	based on pET-24a (*fusA*_wt)	This manuscript
pET-24a (*fusA*_A608E)	based on pET-24a (*fusA*_wt)	This manuscript
pET-24a (*fusA*_A608V)	based on pET-24a (*fusA*_wt)	This manuscript
pET-24a (*fusA*_P610L)	based on pET-24a (*fusA*_wt)	This manuscript
pET-24a (*fusA*_P610Q)	based on pET-24a (*fusA*_wt)	This manuscript
pET-24a (*fusA*_T674A)	based on pET-24a (*fusA*_wt)	This manuscript
pET-24a (*fusA*_A678V)	based on pET-24a (*fusA*_wt)	This manuscript
pET-24a (*fusA*_Y680C)	based on pET-24a (*fusA*_wt)	This manuscript
**Oligonucleotides**		
MF+14Flu mRNA	IBA lifesciences	
**Chemicals, peptides, recombinant proteins**		
cOmplete protease inhibitor	Roche	Cat# 11697498001
DNase I	Thermo Fisher Scientific	Cat# EN0521
Apramycin (Apr)	Sigma-Aldrich	Cat# A2024
Carbenecillin (Car)	Carl Roth	Cat# 6344.3
Chloramphenicol (Cam)	Sigma-Aldrich	Cat# C1919
Dihydrostreptomycin (Dhs)	Sigma-Aldrich	Cat# D7253
Erythromycin (Ery)	Sigma-Aldrich	Cat# 856193
Gentamicin (Gen)	Sigma-Aldrich	Cat# G1264
KanamycinA (KanA)	Sigma-Aldrich	Cat# K4000
Kanamycin B (KanB)	Sigma-Aldrich	Cat# B5264
Kasugamycin (Ksg)	Sigma-Aldrich	Cat# 32354
Neamine (Nea)	Santa Cruz Biotechnology	Cat# sc-338357
NeomycinB (Neo)	Sigma-Aldrich	Cat# N6386
Norfloxacin (Nor)	Sigma-Aldrich	Cat# N9890
Ribostamycin (Rib)	Sigma-Aldrich	Cat# R2255
Sisomicin (Sis)	Sigma-Aldrich	Cat# S7796

Spectinomycin (Spc)	TCI	Cat# SO584
Streptomycin (Str)	Sigma-Aldrich	Cat# S9137
Tetracycline (Tet)	Sigma-Aldrich	Cat# T7660
DTT (Cleland´s reagent, ULTROL)	Merck	Cat# 233153
Iodoacetamide	Sigma-Aldrich	Cat# I1149
Bodipy-FL-succinimidyl ester	Thermo Fisher Scientific	Cat# D6102
Gentamicin Texas Red (GTTR)	AAT Bioquest	Cat# 24300
Isotope-labeled reference peptides	JPT Peptide Technologies	Spike-Tides L
Trypsin (Sequencing Grade modified)	Promega	Cat# V5111
**Critical commercial assays**		
LIVE/DEAD® BacLight™Bacterial Viability Kit	Invitrogen	Cat# L7012
Protino Ni-IDA 2000	Macherey-Nagel	Cat# 745170.25
**Software and algorithms**		
MaxQuant 2.0.1.0 & 2.1.4.0	Cox and Mann (2008) [76]	10.1038/nbt.1511
Perseus 1.6.15.0	Tyanova, Temu, and Cox (2016) [77]	https://maxquant.net/perseus/
PEAKS 10.5	Bioinformatics Solutions Inc.	https://www.bioinfor.com/
Skyline 24.1.0.199	Pino et al. (2020) [78]	10.1002/mas.21540
Spectronaut 17.1.22	Biognosys	https://biognosys.com
Algorithm for the detection of amino acid substitutions	Mordret et al. (2019) [79]	10.1016/j.molcel.2019.06.041
GraphPad PRISM 10	GraphPad Software, Boston	www.graphpad.com
Multi Gauge	FUJIFILM	N/A
MicrobeJ 5.14	Ducret et al. (2016) [80]	https://www.microbej.com/
Fiji 2.14	NIH, Bethesda [81]	https://imagej.nih.gov/ij/
Zen Black 2.3	Zeiss	https://www.zeiss.com/microscopy/us/products/software/zeiss-zen.html
BioRender	BioRender	https://BioRender.com/40melak
CorelDRAW 26.1.0.143	Corel Corporation	https://www.coreldraw.com/de/
CETCORPLUS 4.6.9	CEOS Heidelberg	https://www.ceos-gmbh.de/de
ChimeraX 1.8-1.10.1	Pettersen et al. (2021) [82]	https://www.rbvi.ucsf.edu/chimerax
WinCoot 0.9.8.95	Brown et al. (2015) [83]	https://www2.mrc-lmb.cam.ac.uk/personal/pemsley/coot
CTFFIND-4.1	Rohou and Grigorieff (2015) [84]	https://grigoriefflab.umassmed.edu/ctffind4
EPU 2.3	Thermo Fisher Eindhoven	https://www.thermofisher.com/de/de/home/electron-

		microscopy/products/software-em-3d-vis/software-updates.html
Gautomatch 0.56	Dr. Kai Zhang	https://www.mrc-lmb.cam.ac.uk/kzhang
MotionCor2	Zheng et al. (2017) [85]	https://emcore.ucsf.edu/ucsf-software
PHENIX 1.20.1	Liebschner et al. (2019) [86]	https://www.phenix-online.org
RELION 3.1 & 5.0	Zivanov et al. (2018) [87]	https://www3.mrc-lmb.cam.ac.uk/relion
Xcalibur™ 4.3	Thermo Fisher	
**Other**		
Starion IR/FLA-9000 scanner	FUJIFILM	
FERAstar FS platereader	BMG Labtech	
Orbitrap Exploris 480	Thermo Fisher	
Orbitrap Fusion Lumos Tribrid	Thermo Fisher	
Centrifuges	Beckman Avanti J-30I, JA 25.50 rotor	
Ultracentrifuges	Beckman Optima L-100XP, Ti 50.2 rotor & SW32 rotor	
EmulsiFlex C3	AVESTIN	
LSM880	Zeiss	https://www.zeiss.com/microscopy/us/products/light-microscopes/confocal-microscopes/airyscan.html
Stopped flow apparatus	Applied Photophysics	
**Deposited structural data**		
Structure of C state	Petrychenko et. al. (2021)[54]	PDB: 7PJV
Structure of H1-EFG wt	Petrychenko et. al. (2021)[54]	PDB: 7PJV
Structure of H2-EFG wt	Petrychenko et. al. (2021)[54]	PDB: 7PJW
Structure of C state	This study	EMDB: 55123
Structure of H1 state	This study	EMDB: 55124
Structure of H2 state	This study	EMDB: 55125
Structure of H1-EF-G P610L	This study	PDB: 9RTU; EMDB: 54253
Structure of H2-EF-G P610L	This study	PDB: 9RTV; EMDB: 54254

Supplementary References

1. Wohlgemuth I., *et al.* Translation error clusters induced by aminoglycoside antibiotics. *Nat. Commun.* **12**, 1830 (2021).
2. Sulaiman J. E., Lam H. Proteomic investigation of tolerant *Escherichia coli* populations from cyclic antibiotic treatment. *J. Proteome. Res.* **19**, 900-913 (2020).
3. Porse A., Jahn L. J., Ellabaan M. M. H., Sommer M. O. A. Dominant resistance and negative epistasis can limit the co-selection of de novo resistance mutations and antibiotic resistance genes. *Nat. Commun.* **11**, 1199 (2020).
4. Hoeksema M., Jonker M. J., Brul S., Ter Kuile B. H. Effects of a previously selected antibiotic resistance on mutations acquired during development of a second resistance in *Escherichia coli*. *BMC Genomics* **20**, 284 (2019).
5. Lázár V., *et al.* Bacterial evolution of antibiotic hypersensitivity. *Mol. Syst. Biol.* **9**, 700 (2013).
6. Ghaddar N., Hashemidahaj M., Findlay B. L. Access to high-impact mutations constrains the evolution of antibiotic resistance in soft agar. *Sci. Rep.* **8**, 17023 (2018).
7. Jahn L. J., Munck C., Ellabaan M. M. H., Sommer M. O. A. Adaptive laboratory evolution of antibiotic resistance using different selection regimes lead to similar phenotypes and genotypes. *Front. Microbiol.* **8**, 816 (2017).
8. Ibacache-Quiroga C., Oliveros J. C., Couce A., Blázquez J. Parallel evolution of high-level aminoglycoside resistance in *Escherichia coli* under low and high mutation supply rates. *Front. Microbiol.* **9**, 427 (2018).
9. Hickman R. A., Munck C., Sommer M. O. A. Time-resolved tracking of mutations reveals diverse allele dynamics during *Escherichia coli* antimicrobial adaptive evolution to single drugs and drug pairs. *Front Microbiol* **8**, 893 (2017).
10. Hou Y., Lin Y. P., Sharer J. D., March P. E. *In vivo* selection of conditional-lethal mutations in the gene encoding elongation factor G of *Escherichia coli*. *J. Bacteriol.* **176**, 123-129 (1994).
11. Mogre A., Sengupta T., Veetil R. T., Ravi P., Seshasayee A. S. Genomic analysis reveals distinct concentration-dependent evolutionary trajectories for antibiotic resistance in *Escherichia coli*. *DNA Res.* **21**, 711-726 (2014).
12. Oz T., *et al.* Strength of selection pressure is an important parameter contributing to the complexity of antibiotic resistance evolution. *Mol. Biol. Evol.* **31**, 2387-2401 (2014).
13. Usui M., Yoshii Y., Thiriet-Rupert S., Ghigo J. M., Beloin C. Intermittent antibiotic treatment of bacterial biofilms favors the rapid evolution of resistance. *Commun. Biol.* **6**, 275 (2023).
14. Qi W., Jonker M. J., de Leeuw W., Brul S., Ter Kuile B. H. Reactive oxygen species accelerate de novo acquisition of antibiotic resistance in E. coli. *iScience* **26**, 108373 (2023).
15. Callens M., *et al.* Hypermutator emergence in experimental *Escherichia coli* populations is stress-type dependent. *Evol. Lett.* **7**, 252-261 (2023).
16. Qi W., Jonker M. J., de Leeuw W., Brul S., Ter Kuile B. H. Role of RelA-synthesized (p)ppGpp and ROS-induced mutagenesis in de novo acquisition of antibiotic resistance in E. coli. *iScience* **27**, 109579 (2024).
17. Li X., *et al.* Nonsteroidal anti-inflammatory drug diclofenac accelerates the emergence of antibiotic resistance via mutagenesis. *Environ Pollut* **326**, 121457 (2023).
18. Daruka L., *et al.* ESKAPE pathogens rapidly develop resistance against antibiotics in development in vitro. *Nat. Microbiol.*, (2025).
19. Hernando-Amado S., Sanz-Garcia F., Martinez J. L. Antibiotic Resistance Evolution Is Contingent on the Quorum-Sensing Response in Pseudomonas aeruginosa. *Mol Biol Evol* **36**, 2238-2251 (2019).

20. Scribner M. R., Santos-Lopez A., Marshall C. W., Deitrick C., Cooper V. S. Parallel evolution of tobramycin resistance across species and environments. *mBio* **11**, (2020).
21. Santi I., Manfredi P., Maffei E., Egli A., Jenal U. Evolution of Antibiotic Tolerance Shapes Resistance Development in Chronic Pseudomonas aeruginosa Infections. *mBio* **12**, (2021).
22. Feng Y., Jonker M. J., Moustakas I., Brul S., Ter Kuile B. H. Dynamics of Mutations during Development of Resistance by Pseudomonas aeruginosa against Five Antibiotics. *Antimicrob Agents Chemother* **60**, 4229-4236 (2016).
23. Sanz-García F., Hernando-Amado S., Martínez J. L. Mutational evolution of *Pseudomonas aeruginosa* resistance to ribosome-targeting antibiotics. *Front. Genet.* **9**, 451 (2018).
24. Bolard A., Plésiat P., Jeannot K. Mutations in gene *fusA1* as a novel mechanism of aminoglycoside resistance in clinical strains of *Pseudomonas aeruginosa*. *Antimicrob. Agents Chemother.* **62**, (2018).
25. Rodriguez de Evgrafov M. C., Faza M., Asimakopoulos K., Sommer M. O. A. Systematic investigation of resistance evolution to common antibiotics reveals conserved collateral responses across common human pathogens. *Antimicrob. Agents Chemother.* **65**, (2020).
26. Ramsay K. A., McTavish S. M., Wardell S. J. T., Lamont I. L. The Effects of Sub-inhibitory Antibiotic Concentrations on Pseudomonas aeruginosa: Reduced Susceptibility Due to Mutations. *Front Microbiol* **12**, 789550 (2021).
27. Laborda P., Martínez J. L., Hernando-Amado S. Convergent phenotypic evolution towards fosfomycin collateral sensitivity of *Pseudomonas aeruginosa* antibiotic-resistant mutants. *Microb. Biotechnol.* **15**, 613-629 (2022).
28. Maunders E. A., Triniman R. C., Western J., Rahman T., Welch M. Global reprogramming of virulence and antibiotic resistance in *Pseudomonas aeruginosa* by a single nucleotide polymorphism in elongation factor, *fusA1*. *J. Biol. Chem.* **295**, 16411-16426 (2020).
29. Abisado-Duque R. G., *et al.* An Amino Acid Substitution in Elongation Factor EF-G1A Alters the Antibiotic Susceptibility of Pseudomonas aeruginosa LasR-Null Mutants. *J Bacteriol* **205**, e0011423 (2023).
30. Abisado R. G., *et al.* Tobramycin Adaptation Enhances Policing of Social Cheaters in Pseudomonas aeruginosa. *Appl Environ Microbiol* **87**, e0002921 (2021).
31. Wardell S. J. T., Rehman A., Martin L. W., Winstanley C., Patrick W. M., Lamont I. L. A large-scale whole-genome comparison shows that experimental evolution in response to antibiotics predicts changes in naturally evolved clinical Pseudomonas aeruginosa. *Antimicrob Agents Chemother* **63**, (2019).
32. Thacharodi A., Lamont I. L. Gene-gene interactions reduce aminoglycoside susceptibility of *Pseudomonas aeruginosa* through efflux pump-dependent and -independent mechanisms. *Antibiotics (Basel)* **12**, (2023).
33. Vestergaard M., Paulander W., Leng B., Nielsen J. B., Westh H. T., Ingmer H. Novel Pathways for Ameliorating the Fitness Cost of Gentamicin Resistant Small Colony Variants. *Front Microbiol* **7**, 1866 (2016).
34. Cao S., Huseby D. L., Brandis G., Hughes D. Alternative Evolutionary Pathways for Drug-Resistant Small Colony Variant Mutants in Staphylococcus aureus. *mBio* **8**, (2017).
35. Heidarian S., Guliaev A., Nicoloff H., Hjort K., Andersson D. I. High prevalence of heteroresistance in Staphylococcus aureus is caused by a multitude of mutations in core genes. *PLoS Biol* **22**, e3002457 (2024).
36. Ma S., *et al.* Mechanisms of Staphylococcus aureus Antibiotics Resistance Revealed by Adaptive Laboratory Evolution. *Curr Microbiol* **82**, 46 (2025).

37. Lozano-Huntelman N. A., *et al.* Evolution of antibiotic cross-resistance and collateral sensitivity in *Staphylococcus epidermidis* using the mutant prevention concentration and the mutant selection window. *Evol. Appl.* **13**, 808-823 (2020).
38. Boyd S. M., *et al.* Genomic characterization of antibiotic-resistant Staphylococcus epidermidis with observed shifts in optimal temperature. *J Appl Microbiol* **135**, (2024).
39. Holley C. L., *et al.* A single amino acid substitution in elongation factor G can confer low-level gentamicin resistance in *Neisseria gonorrhoeae*. *Antimicrob. Agents Chemother.* **66**, e0025122 (2022).
40. Golparian D., Jacobsson S., Holley C. L., Shafer W. M., Unemo M. High-level in vitro resistance to gentamicin acquired in a stepwise manner in Neisseria gonorrhoeae. *J Antimicrob Chemother* **78**, 1769-1778 (2023).
41. Waller N. J. E., Cheung C. Y., Cook G. M., McNeil M. B. The evolution of antibiotic resistance is associated with collateral drug phenotypes in *Mycobacterium tuberculosis*. *Nat. Commun.* **14**, 1517 (2023).
42. Kuhberger R., Piepersberg W., Petzet A., Buckel P., Bock A. Alteration of ribosomal protein L6 in gentamicin-resistant strains of Escherichia coli. Effects on fidelity of protein synthesis. *Biochemistry* **18**, 187-193 (1979).
43. Wasserman M. R., *et al.* Chemically related 4,5-linked aminoglycoside antibiotics drive subunit rotation in opposite directions. *Nat. Commun.* **6**, 7896 (2015).
44. Hobbie S. N., *et al.* Binding of neomycin-class aminoglycoside antibiotics to mutant ribosomes with alterations in the A site of 16S rRNA. *Antimicrob. Agents Chemother.* **50**, 1489-1496 (2006).
45. Bollen A., Cabezon T., de Wilde M., Villarroel R., Herzog A. Alteration of ribosomal protein S17 by mutation linked to neamine resistance in Escherichia coli. I. General properties of neaA mutants. *J Mol Biol* **99**, 795-806 (1975).
46. Yaguchi M., *et al.* Alteration of ribosomal protein S17 by mutation linked to neamine resistance in Escherichia coli. II. Localization of the amino acid replacement in protein S17 from a meaA mutant. *J Mol Biol* **104**, 617-620 (1976).
47. Yaguchi M., *et al.* Cooperative control of translational fidelity by ribosomal proteins in Escherichia coli. II. Localization of amino acid replacements in proteins S5 and S12 altered in double mutants resistant to neamine. *Mol Gen Genet* **142**, 35-43 (1975).
48. Peske F., Savelsbergh A., Katunin V. I., Rodnina M. V., Wintermeyer W. Conformational changes of the small ribosomal subunit during elongation factor G-dependent tRNA-mRNA translocation. *J. Mol. Biol.* **343**, 1183-1194 (2004).
49. Safdari H. A., *et al.* The translation inhibitors kasugamycin, edeine and GE81112 target distinct steps during 30S initiation complex formation. *Nat Commun* **16**, 2470 (2025).
50. Zhang Y., *et al.* The context of the ribosome binding site in mRNAs defines specificity of action of kasugamycin, an inhibitor of translation initiation. *Proc. Natl. Acad. Sci. USA.* **119**, (2022).
51. Bilgin N., Richter A. A., Ehrenberg M., Dahlberg A. E., Kurland C. G. Ribosomal RNA and protein mutants resistant to spectinomycin. *EMBO J* **9**, 735-739 (1990).
52. Kamath D., Gregory S. T., O'Connor M. The Loop 2 Region of Ribosomal Protein uS5 Influences Spectinomycin Sensitivity, Translational Fidelity, and Ribosome Biogenesis. *Antimicrob Agents Chemother* **61**, (2017).
53. Shapovalova K. S., *et al.* Synthesis and antibacterial activity of new 6''-modified tobramycin derivatives. *Antibiotics (Basel)* **13**, (2024).
54. Petrychenko V., Peng B. Z., de A. P. Schwarzer A. C., Peske F., Rodnina M. V., Fischer N. Structural mechanism of GTPase-powered ribosome-tRNA movement. *Nat. Commun.* **12**, 5933 (2021).
55. Paternoga H., *et al.* Structural conservation of antibiotic interaction with ribosomes. *Nat Struct Mol Biol* **30**, 1380-1392 (2023).

56. Taber H. W., Mueller J. P., Miller P. F., Arrow A. S. Bacterial uptake of aminoglycoside antibiotics. *Microbiol. Rev.* **51**, 439-457 (1987).
57. Davis B. D. Mechanism of bactericidal action of aminoglycosides. *Microbiol. Rev.* **51**, 341-350 (1987).
58. Baquero F., Levin B. R. Proximate and ultimate causes of the bactericidal action of antibiotics. *Nat Rev Microbiol* **19**, 123-132 (2021).
59. Seupt A., Schniederjans M., Tomasch J., Häussler S. Expression of the MexXY aminoglycoside efflux pump and presence of an aminoglycoside-modifying enzyme in clinical *Pseudomonas aeruginosa* isolates are highly correlated. *Antimicrob. Agents Chemother.* **65**, (2020).
60. Macvanin M., Ballagi A., Hughes D. Fusidic acid-resistant mutants of Salmonella enterica serovar typhimurium have low levels of heme and a reduced rate of respiration and are sensitive to oxidative stress. *Antimicrob Agents Chemother* **48**, 3877-3883 (2004).
61. Macvanin M., Johanson U., Ehrenberg M., Hughes D. Fusidic acid-resistant EF-G perturbs the accumulation of ppGpp. *Mol Microbiol* **37**, 98-107 (2000).
62. Das B., Bhadra R. K. (p)ppGpp metabolism and antimicrobial resistance in bacterial pathogens. *Front. Microbiol.* **11**, 563944 (2020).
63. Li W.*, et al.* Mechanism of tetracycline resistance by ribosomal protection protein Tet(O). *Nat Commun* **4**, 1477 (2013).
64. Lang M., Carvalho A., Baharoglu Z., Mazel D. Aminoglycoside uptake, stress, and potentiation in Gram-negative bacteria: new therapies with old molecules. *Microbiol. Mol. Biol. Rev.* **87**, e0003622 (2023).
65. Rosenberg E. Y., Ma D., Nikaido H. AcrD of *Escherichia coli* is an aminoglycoside efflux pump. *J. Bacteriol.* **182**, 1754-1756 (2000).
66. Kumar A., Schweizer H. P. Bacterial resistance to antibiotics: active efflux and reduced uptake. *Adv. Drug Del. Rev.* **57**, 1486-1513 (2005).
67. Hinz A., Lee S., Jacoby K., Manoil C. Membrane proteases and aminoglycoside antibiotic resistance. *J. Bacteriol.* **193**, 4790-4797 (2011).
68. Goltermann L., Good L., Bentin T. Chaperonins fight aminoglycoside-induced protein misfolding and promote short-term tolerance in Escherichia coli. *J Biol Chem* **288**, 10483-10489 (2013).
69. Goltermann L., Sarusie M. V., Bentin T. Chaperonin GroEL/GroES Over-Expression Promotes Aminoglycoside Resistance and Reduces Drug Susceptibilities in Escherichia coli Following Exposure to Sublethal Aminoglycoside Doses. *Front Microbiol* **6**, 1572 (2015).
70. Ling J., Cho C., Guo L. T., Aerni H. R., Rinehart J., Söll D. Protein aggregation caused by aminoglycoside action is prevented by a hydrogen peroxide scavenger. *Mol. Cell* **48**, 713-722 (2012).
71. Gao Y. G., Selmer M., Dunham C. M., Weixlbaumer A., Kelley A. C., Ramakrishnan V. The structure of the ribosome with elongation factor G trapped in the posttranslocational state. *Science* **326**, 694-699 (2009).
72. Tyanova S., Temu T., Cox J. The MaxQuant computational platform for mass spectrometry-based shotgun proteomics. *Nat. Protoc.* **11**, 2301-2319 (2016).
73. Gessulat S.*, et al.* Prosit: proteome-wide prediction of peptide tandem mass spectra by deep learning. *Nat. Methods* **16**, 509-518 (2019).
74. Pino L. K., Searle B. C., Bollinger J. G., Nunn B., MacLean B., MacCoss M. J. The Skyline ecosystem: Informatics for quantitative mass spectrometry proteomics. *Mass. Spectrom. Rev.* **39**, 229-244 (2020).
75. Kramer E. B., Farabaugh P. J. The frequency of translational misreading errors in E. coli is largely determined by tRNA competition. *RNA* **13**, 87-96 (2007).

76. Cox J., Mann M. MaxQuant enables high peptide identification rates, individualized p.p.b.-range mass accuracies and proteome-wide protein quantification. *Nat. Biotechnol.* **26**, 1367-1372 (2008).
77. Tyanova S.*, et al.* The Perseus computational platform for comprehensive analysis of (prote)omics data. *Nat. Methods* **13**, 731-740 (2016).
78. Pino L. K., Searle B. C., Bollinger J. G., Nunn B., MacLean B., MacCoss M. J. The Skyline ecosystem: Informatics for quantitative mass spectrometry proteomics. *Mass Spectrom. Rev.* **39**, 229-244 (2020).
79. Mordret E.*, et al.* Systematic detection of amino acid substitutions in proteomes reveals mechanistic basis of ribosome errors and selection for translation fidelity. *Mol. Cell* **75**, 427-441 e425 (2019).
80. Ducret A., Quardokus E. M., Brun Y. V. MicrobeJ, a tool for high throughput bacterial cell detection and quantitative analysis. *Nat. Microbiol.* **1**, 16077 (2016).
81. Schindelin J.*, et al.* Fiji: an open-source platform for biological-image analysis. *Nat. Methods* **9**, 676-682 (2012).
82. Pettersen E. F.*, et al.* UCSF ChimeraX: Structure visualization for researchers, educators, and developers. *Protein Sci.* **30**, 70-82 (2021).
83. Brown A., Long F., Nicholls R. A., Toots J., Emsley P., Murshudov G. Tools for macromolecular model building and refinement into electron cryo-microscopy reconstructions. *Acta Crystallogr. D Biol. Crystallogr.* **71**, 136-153 (2015).
84. Rohou A., Grigorieff N. CTFFIND4: Fast and accurate defocus estimation from electron micrographs. *J. Struct. Biol.* **192**, 216-221 (2015).
85. Zheng S. Q., Palovcak E., Armache J. P., Verba K. A., Cheng Y., Agard D. A. MotionCor2: anisotropic correction of beam-induced motion for improved cryo-electron microscopy. *Nat. Methods* **14**, 331-332 (2017).
86. Liebschner D.*, et al.* Macromolecular structure determination using X-rays, neutrons and electrons: recent developments in Phenix. *Acta Crystallogr. D Struct. Biol.* **75**, 861-877 (2019).
87. Zivanov J.*, et al.* New tools for automated high-resolution cryo-EM structure determination in RELION-3. *Elife* **7**, (2018).
