# Supplementary material for: Selective silencing of antibiotic-tethered ribosomes as a resistance mechanism against aminoglycosides
Source: Nat Commun. 2025 Oct 29;16:9568. doi: 10.1038/s41467-025-65298-7 (PMC12572303; doi:10.1038/s41467-025-65298-7)
Supplement: Supplementary file 2 — Description of Additional Supplementary Files [file 41467_2025_65298_MOESM2_ESM.pdf]

Description of Additional Supplementary Files:

Supplementary Data 1: EF-G resistance variants across pathogens: Amino acid substitutions in EF-G from diverse pathogens that evolved under aminoglycoside selection (blue) or were identified in clinical isolates (red). Corresponding aminoglycosides and PubMed references are listed. The sequences are aligned to the EF-G sequence from *E. coli* MG1655.
